# Supplementary material for: The spatial relation of diabetic retinal neurodegeneration with diabetic retinopathy
Source: PLoS One. 2020 Apr 16;15(4):e0231552. doi: 10.1371/journal.pone.0231552 (PMC7161968; doi:10.1371/journal.pone.0231552)
Supplement: Attachment — Submitted filename: Response to Reviewers.docx [file pone.0231552.s002.docx]

*Dear editor,
Dear reviewer(s),

Thank you for the opportunity to revise our manuscript, and reply in more detail to some of the questions it raised. Your thorough review and constructive suggestions are very much appreciated.

Below you may find the comments of the reviewer with our responses in blue.*

*Thank you for your consideration.*

*Sincerely,*

*Aleid van de Kreeke, MD*

Reviewer 1

The authors present an interesting analysis of structural changes in time series of OCT data from diabetic patients. The aim of their work is clearly expressed. In general, the statistical analysis and the methodology is correct.

We would like to thank the reviewer for the appreciation of our manuscript.

I have some concerns regarding the multilevel modelling:

- From their description at lines 151-153 it would look like they have employed 4 nested factors (quadrants, eye, visits and patient). However, quadrants and visits are effectively fixed effects in the analysis (calculations per quadrants are clearly reported in the table and the visit is effectively the same as the time variable in the analysis). The only two random effects should therefore only be patient and eye (nested)

In the analysis we considered four levels: repeated measures, quadrants, eyes and patients. In the analyses, therefore, we included random intercepts for quadrants, eyes and patients. The reviewer is absolutely right that a random intercept for quadrants is more or less the same as adding quadrant as variable to the multilevel model. In some of the analyses, the random intercept variance on quadrant level indeed reduced to zero. However, in some of the analyses, the random intercept variance on quadrant level decreased, but remained relevant, so in those models we decided to leave the random intercept on quadrant level in the model. Because time was the lowest level in the analysis, there was no random intercept on time.

- Were random slopes included in the analysis? Since each eye represents a different time series, having random slopes specific for each eye would greatly improve the accuracy of the estimates

Although the reviewer is absolutely right, we did not include random slopes to the models. This was done because of the complexity of the models, and our experience that adding random slopes to complicated models (sometimes) lead to unstable estimates.

One of the stated aims (and claimed results in the discussion) is to understand whether structural changes are associated with the (local) development of DR. The authors seem to imply in their discussion that they have gathered evidence for thinning of the inner retina as a prognostic factor for the development of DR. This is evident when they draw the parallelism with similar studies investigating the prognostic ability of functional measurements. However, their analysis does not focus on an accurate assessment of the risk of developing DR given the structural parameters. Indeed, their results, as presented, would be more correctly interpreted as faster loss of inner retinal neurons as a consequence of DR. The authors are unclear about what interpretation they want to support and this should be clarified.

Also, there is no mention of how many subjects (sectors?) actually converted to DR during the study. If the aim is to show that neural loss is associated with an increased risk of developing DR, or simply micro-vascular changes in a given sector, an analysis with a logistic regression (or better yet, a survival analysis able to incorporate the temporal dimension) should be performed using the structural parameters as a predictor and the conversion to DR as the response variable.

Both these (valid) points are very much related, which is why we will answer them as one. Our original set-up of the study was intended as the reviewer already suggests: the analysis of conversion of retinal quadrants during the study to early DR. We then wanted to analyze if these retinal quadrants specifically showed an increased neuroretinal degeneration prior to this conversion. Unfortunately, during the analyses of the results we encountered two major problems, making such analyses impossible:

1) The development of DR turned out to not simply be a ‘conversion’. Several quadrants/subjects had, for example, no DR at baseline, but developed DR at visit 2, which subsequently was again gone at visit 3 and 4. Indeed, there were even quadrants/subjects showing DR at baseline, which disappeared during the follow-up period. Pretty much any pattern of variable DR expression throughout the follow-up period was found. As such, we were unable to simply classify our subjects/quadrants into the three logical groups: ‘no DR’, ‘development of DR’ or ‘DR present throughout’.
2) When realizing the problem mention above, we decided to create 2 extra groups: 1) DR at baseline or some other visit which was not present at the final visit and 2) increase of minor DR over time. This gave us a total of 5 different groups, some with a very low N. Statistical analyses using these groups became too unreliable to interpret the results. Even when trying to eliminate 1 or 2 groups we kept encountering this problem. As such, we decided to dichotomize the quadrants/subjects into the 2 groups now used in this paper: no DR throughout, and (minor) DR either already present or developed at some point during the follow-up period.

We believe that although this makes a claim of ‘structural changes lead to DR development’ impossible, it is still provides evidence to the relatedness of both these changes in the retina of diabetics. As we looked at a very local (quadrant) level, we feel we are able to claim that local structural changes are related to local vascular changes. It is, however, true that we are unable to provide evidence that structural changes lead to vascular changes. We can merely illustrate a local association between the two. As our current manner of describing our findings in the discussion of our paper can apparently be misleading (as the reviewer interpreted this slightly different than we intended), we decided to change some sentences/sections in the discussion section. The following was changed:

- Page 11, line 246: *“… and vice versa…”* is added
- Page 11, line 255-256: *“…also constitutes…”* is replaced by *“…is associated with…”*
- Page 11, line 257: *“…tend…”* is replaced with *“…seem…”*
- Page 12, line 281: *“…of…”* is replaced with *“…between…”*

Reviewer 2

In this paper the relationship between diabetes-induced retinal neurodegeneration and microvascular changes in fundus photography is examined in a prospective observational study. The main conclusion is that RNFL and GCL showed significant thinning over time and these changes were more pronounced with the presence or development of DR. Although a control group is lacking, this is a very interesting study which add new clinical data regarding the relationship between retinal neurodegeneration and the development microvascular abnormalities in the setting of diabetic retinopathy.

We would like to thank the reviewer for the appreciation of our manuscript.

There are, however, several issues that should be addressed:

In the introduction and/or in the discussion, the results of EUROCONDOR study in which two phenotypes of early stages of DR: one with diabetes induced retinal neurodegeneration + microvascular abnormalities, and another in which microvascular abnormalities were detected without any sign of neurodegeneration should be mentioned. In this regard, the authors could comment on the percentage of patients who developed microvascular abnormalities without significant thinning in RNFL and/or GCL in SD-OCT.

This is a very valid point, overlooked from our side. In over 30% of patients of the Eurocondor study, no neuroretinal degeneration (defined as a retinal layer thickness measurement 2SD below the normative mean) was seen in patients with signs of DR. In the present study we analyzed the change in retinal layer thickness over time, for which there is no normative database. Therefore, we were unable to proof the assumption that the decrease in retinal layer thickness due to diabetes mellitus is greater than the physiological decrease in retinal layer thickness with age. Nevertheless, the decrease in retinal layer thickness is highest in areas with DR. As this is indeed a very relevant paper to our study, we have added the following sections to both the introduction and discussion:

- Page 4, line 70: *“It also has to be noted that the EUROCONDOR study found that, while there was indeed a clear association between DRN and vasculopathy, there is also a significant amount of diabetic patients not expressing this association.”* was added.
- Page 11, line 248: *“However, the EUROCONDOR study did find that approximately 1 out of 3 diabetic patients (32%) do not express DRN despite the presence of minor DR, suggesting that while there seems to be a link in the pathogenesis of the two, this is not the same for all patients”* was added.

The progression of microvascular abnormalities during follow-up and their relationship with neurodegeneration should be better analyzed. A separate analysis between new incident microvascular lesions and those already present at the beginning of the study could clarify the role of neurodegeneration on new onset of vascular abnormalities (at least in a subset of patients). The question of whether or not neurodegeneration can predict the appearance of microvascular abnormalities in the natural history of DR should be addressed by the authors taken into account the prospective nature of this study.

While this is a valid point also raised by reviewer #1, we were unfortunately unable to perform the analysis as mentioned. The reason for this is also outlined above, but will be repeated here:

Our original set-up of the study was intended as the reviewer already suggests: the analysis of conversion of retinal quadrants during the study to early DR. We then wanted to analyze if these retinal quadrants specifically showed an increased neuroretinal degeneration prior to this conversion. Unfortunately, during the analyses of the results we encountered two major problems, making such analyses impossible:

1) The development of DR turned out to not simply be a ‘conversion’. Several quadrants/subjects had, for example, no DR at baseline, but developed DR at visit 2, which subsequently was again gone at visit 3 and 4. Indeed, there were even quadrants/subjects showing DR at baseline, which disappeared during the follow-up period. Pretty much any pattern of variable DR expression throughout the follow-up period was found. As such, we were unable to simply classify our subjects/quadrants into the three logical groups: ‘no DR’, ‘development of DR’ or ‘DR present throughout’.
2) When realizing the problem mention above, we decided to create 2 extra groups: 1) DR at baseline or some other visit which was not present at the final visit and 2) increase of minor DR over time. This gave us a total of 5 different groups, some with a very low N. Statistical analyses using these groups became too unreliable to interpret the results. Even when trying to eliminate 1 or 2 groups we kept encountering this problem. As such, we decided to dichotomize the quadrants/subjects into the 2 groups now used in this paper: no DR throughout, and (minor) DR either already present or developed at some point during the follow-up period.

The second sentence of the first paragraph of the discussion (lines 203-204) needs to be completed with appropriated references.

We have added several references supporting the statement.

Apart from vascular leakage, glial activation could also contribute to the thinning of IPL. The authors perhaps could add a comment on this issue to the discussion section.

While we feel that this may indeed play a role purely on a theoretical basis (i.e. glial activation due to early inflammatory processes may result in an increase in volume of these glial cells, resulting in a very slight thickening of certain retinal layers), we were unable to find literature supporting this statement. Yet we decided to add the following sentence nonetheless, as per suggestion from the reviewer:

- Page 11, line 238: *“Furthermore, IPL thickening may also be caused by glial cell activation due to early inflammation, increasing the volume of these cells and therefore ultimately the volume of certain retinal layers.”* was added.
