# Supplementary material for: The spatial relation of diabetic retinal neurodegeneration with diabetic retinopathy
Source: PLoS One. 2020 Apr 16;15(4):e0231552. doi: 10.1371/journal.pone.0231552 (PMC7161968; doi:10.1371/journal.pone.0231552)
Supplement: Attachment — Submitted filename: Response to Reviewers 2.docx [file pone.0231552.s003.docx]

*Dear editor,
Dear reviewer(s),

Thank you for the opportunity to once more revise our manuscript, and reply in more detail to some of the questions it raised. Your thorough review and constructive suggestions are very much appreciated.

Below you may find the last comments of the reviewer with our responses in blue.*

*Thank you for your consideration.*

*Sincerely,*

*Aleid van de Kreeke, MD*

Reviewer 1

I appreciate that the authors provided explanation to some of my concerns. To my eyes, however, some sentences still read as confusing:

- Lines 245 - 246: "...thus making increased neurodegeneration a sign of potential worsening of retinal vascular status and vice versa in DM" should be removed

The sentence has been removed

- Lines 255 - 258: "This means that locally increased DRN is associated with also constitutes an increased risk/presence for (development of) DR locally, i.e.: quadrants that suffer from increased DRN tend seem to develop or suffer from DR more often, regardless of the overall DR status of that eye as a whole." should be reworded to avoid suggesting a causative effect. The word "development" is especially misleading.

We changed the sentence into what we hope is somewhat less misleading, by removing the words ‘development’ and ‘develop’. The sentence now reads as follows (page 11, lines 254-257): *“This means that locally increased DRN is associated with an increased risk/presence of DR locally, i.e.: quadrants that suffer from increased DRN seem to suffer from DR more often, regardless of the overall DR status of that eye as a whole.”*

Finally, I am still convinced that the sector should not be a random effect. The author explicitly use the different sectors to make inference from direct comparisons, reporting p-values. This is exactly when a factor should be a fixed rather than a random effect. Moreover, having the same factor twice (as random and fixed effect) generates collinearity and is simply wrong. Numerical improvement of fitting should not replace good reasoning when building statistical models.

You are absolutely right. Adding the variable to the fixed part of the model is comparable to adding a random intercept for that particular variable. However, due to the four level structure of the data the fixed regression coefficients for the quadrants are a sort of average difference between the quadrants, while the random part reflects the differences between the quadrants within the eyes/patients. Because this is slightly different, a random intercept for quadrant still does make sense and therefore it is possible to add both to the multilevel model. However, if the reviewer insists we can leave the quadrant level out of the multilevel model. It will probably not lead to big changes in the results and conclusions.
