# Supplementary material for: The spatial relation of diabetic retinal neurodegeneration with diabetic retinopathy
Source: PLoS One. 2020 Apr 16;15(4):e0231552. doi: 10.1371/journal.pone.0231552 (PMC7161968; doi:10.1371/journal.pone.0231552)
Supplement: Attachment — Submitted filename: Response to Reviewers 3.docx [file pone.0231552.s004.docx]

*Dear editor,
Dear reviewer(s),

Thank you for the opportunity to once more revise our manuscript, and reply in more detail to some of the questions it raised. Your thorough review and constructive suggestions are very much appreciated.

Below you may find the last comments of the reviewer with our responses in blue.*

*Thank you for your consideration.*

*Sincerely,*

*Aleid van de Kreeke, MD*

Reviewer 1

The authors have addressed all my concerns except the ones regarding the random effects in the mixed model. I appreciate the explanation, but what they have done does not account for differences between quadrants in the same subject/eye. If they wanted to model this, they should have used a random SLOPE for the effect of quadrants. This would model the INDIVIDUAL effect of quadrants within each eye/individual.

Allow me to elaborate with a simpler example (Quadrant is a discrete factor in the model):

y = B0 + B1*Quadrant + (1|Subject/Eye) #This models the quadrant as a fixed effect

y = B0 + B1*Quadrant + (1|Subject/Eye/Quadrant) #This models the quadrant as a fixed effect and random intercept, nested within the eye. This model shows a bad use of random effects, since the grouping factor is considered both as a random and a fixed effect.

y = B0 + B1*Quadrant + (1|Quadrant/Subject/Eye) #This is useless

y = B0 + B1*Quadrant + (1|Subject/Eye) + (1|Quadrant) #This is equally useless

y = B0 + B1*Quadrant + (Quadrant|Subject/Eye) #THIS models individual effects of the quadrant within each eye/subject, which is what the authors state as their goal for including the quadrant in the random effects. In this case, Quadrant is a random slope on the fixed effect.

Due to the concerns raised by the reviewer, we decided to remove the quadrant level. Tables 1 and 2, as well as figure 2, have been updated with the results obtained with the new analyses (i.e. analyses without the quadrant level). The methods section has also been adjusted accordingly (page 7, lines 153-154). As the results did not change drastically, the message our paper conveys did not change either, and therefore the discussion and conclusions required no alteration.
